# Population-Based Study of Birth Prevalence and Factors Associated with Cleft Lip and/or Palate in Taiwan 2002–2009

**DOI:** 10.1371/journal.pone.0058690

**Published:** 2013-03-26

**Authors:** Ruoh-Lih Lei, Huey-Shys Chen, Bao-Yuan Huang, Yueh-Chih Chen, Philip Kuo-Ting Chen, Huei-Ying Lee, Chi-Wen Chang, Chih-Lung Wu

**Affiliations:** 1 Department of Nursing, College of Medicine and Nursing, HungKuang University, Taiwan, Republic of China; 2 Department of Health Promotion, Outcomes, Systems, and Policy, Doctor of Nursing Practice Program, College of Nursing, University of Toledo, Toledo, Ohio, United States of America; 3 Department Of Childhood Education and Enterprise Management, Central Taiwan University of Science and Technology, Taiwan, Republic of China; 4 Craniofacial Center, Chang Gung Memorial Hospital, Chang Gung University, Taoyuan, Taiwan, Republic of China; 5 Department of Nursing, College of Nursing, Kaohsiung Medical University, Taiwan, Republic of China; 6 Department of Nursing, College of Medicine, Chang Gung University, Taoyuan, Taiwan, Republic of China; 7 Department of Orthopedic Surgery, Chung Shan Medical University Hospital, School of Medicine, Chung Shan Medical University, Taichung, Taiwan, Republic of China; University of Montreal, Canada

## Abstract

**Background:**

Facial cleft deformities, including cleft lip with or without cleft palate (CL/P) and cleft palate (CP), are common congenital birth anomalies, especially in Asia. This study aimed to analyze the prevalence of CL/P and CP and to identify associated factors in Taiwan.

**Methods:**

This population-based epidemiological study retrospectively analyzed birth data obtained from the Department of Health in Taiwan for years 2002–2009. Frequency distribution, percentages and related predictors were investigated, and findings were presented by types of cleft deformities. Logistic regression analysis was performed to identify factors associated with cleft deformities.

**Results:**

Overall prevalence of cleft deformities among 1,705,192 births was 0.1% for CL/P and 0.04% for CP over the 8-year study period. Higher prevalence of CL/P or CP was observed with multiple pregnancies, being male for CL/P, being female for CP, gestational age ≤37 weeks and lower birth weight (<1.5 kg). Both CL/P and CP were significantly associated with gestational age <37 weeks and birth weight<1.5 kg (all P <0.0001). CL/P was significantly associated with multiple parities (P = 0.0004–0.002). Male newborns and female newborns were significantly associated with CL/P and CP, respectively (both P<0.0001).

**Conclusions:**

Overall prevalence for congenital cleft deformities in study subjects was 0.1%, in keeping with high rates in Asia. Results suggest the need for awareness and early identification of those at high risk for cleft deformities, including newborns with gestational age <37 weeks, weighing <1.5 kg at birth and women with multiple parities, as a potential strategy to counter long-term adverse effects on speech and language in this population.

## Introduction

Facial cleft deformities, including cleft lip with or without cleft palate (CL/P) and cleft palate (CP), are among the most common congenital birth anomalies. While the worldwide prevalence of such deformities is about 1.5 per 1,000 live births, the rate varies six-fold for CL/P and three-fold for CP [1]. Reports in Asian populations put overall rates around 1.76 to 1.81, reflecting the higher prevalence in this region [2], [3]. A report characterized the prevalence of CL/P and CP at 2.3 per 1,000 newborns in Taiwan in 2000 [4], which is somewhat higher than that reported in other Asian populations, although methods of tabulation vary [2], [3], [5]. A recent study in a tertiary hospital in Taiwan found a birth rate of 3.2 facial cleft deformities per 1,000 newborns, a rate likely reflecting the status of the hospital as a referral center for high risk births [6].

Even when newborns with cleft deformities receive appropriate treatment, some still have facial deformity and speech impairment [7]–[9], which further increases the health care and familial burden of the disease [10]. Asians, boys in particular, experience greater psychosocial injury from cleft deformities than that found in whites studied in the US and Europe [11]. However, the Center for Disease Control, Department of Health, has not reported the birth prevalence of CL/P or CP in Taiwan since 2000, when it ceased issuing annual reports [4]. Worldwide, the World Health Organization and the International Perinatal Database of Typical Oral Clefts Working Group have called for efforts to characterize rates of facial cleft deformities and related factors [1], [12], [13]. Therefore, we sought to characterize the birth prevalence of CL/P and CP in Taiwan from 2002 to 2009, and to identify factors that may be associated with their prevalence over time.

## Methods

We conducted a population-based epidemiologic study utilizing retrospective secondary database analysis of national data collected by the Taiwan Bureau of Health Promotion of the Department of Health, Executive Yuan from 2002 to 2009. The Executive Yuan collects health information from all hospitals, as well as other providers, to help guide the administration of public health policy in Taiwan. For this analysis, we used data from the Birth Registration Database [14], which were collected from a total of 1,705,192 births over the eight years. Data analyzed include number of live births, stillbirths, nationality of mother and spouse, gender of neonate, gestational age at birth (in weeks), birth weight (g), parity (first child, second, other) and congenital defects (CL/P (cleft lip with or without cleft palate: [code 0203]; CP (no cleft lip): [code 0204]; other congenital anomalies). Only live births were included in analysis. This study conformed to the ethical standards of the responsible committee on human experimentation (institutional or regional) and with the Helsinki Declaration of 1975, as revised in 2004 and was approved by the Institutional Review Board of the Hung Kuang University. As the data were de-identified, informed consent of subjects was not required.

### Statistical analysis

Statistical analyses was done to examine the correlation of demographic characteristics of mothers and newborns with CL/P and CL. To examine the effect over time, the entire duration was divided into earlier and later periods (2002–2005 and 2006–2009), and the distribution of CL/P and CP was compared between the two groups using Chi-square test; data analyzed included maternal age (<34 years, ≥34 years), parity (first birth, second, other), newborn gender, gestational age (<37 weeks, ≥37 weeks) and birth weight (<1.5 kg, 1.5–2.5 kg, >2.5 kg). Count data were described using frequency and percentage. Logistic regression analysis was applied to identify factors associated with CL/P and CP. Statistical analysis was performed with SPSS version 18.0 software (SPSS, Inc., Chicago, IL). A p value of <0.05 was established as statistical significance.

## Results

The demographic and clinical characteristics of mothers and newborns are presented in Table 1. Birth prevalence of CL/P, CP and other congenital anomalies for newborns in Taiwan from 2002 to 2009 are presented in Table 2. Data were collected from a total of 1,705,192 births over the eight years of analysis. The number of births declined for each year, from 243,801 in 2002 to 194,489 in 2009. The 14,111 newborns with major congenital anomalies of all types accounted for 0.8% of newborns. Of these, 2,352 had CL/P or CP, accounting for 0.1% of newborns and 16.7% of those with congenital anomalies. The 690 newborns with CP accounted for 0.04% of newborns and 4.9% of those with congenital anomalies. Analysis showed the highest prevalence (0.1%) of CL/P in 2002 with 271 newborns. The highest birth prevalence (0.05%) of CP was in 2003 with 109 infants. The lowest birth prevalence of cleft abnormalities (0.1%) was in 2009 when 154 infants were diagnosed with CL/P (0.08%) and 70 (0.04%) with CP. Analysis of the change in prevalence of CL/P and CP in Taiwan 2002–2009 showed that the combined prevalence of CL/P and CP in Taiwan declined from 2005 (Table 2). Notably, the odds of CL/P after 2006 is 1.2 (95%CI: 1.1–1.3, P<0.001, data not shown) times higher than the odds before 2005, while no such observation is noted in newborns with CP (P = 0.5).

**Table 1:** Demographic and clinical characteristics of mothers and newborns.

	Years									
	2002	2003	2004	2005	2006	2007	2008		2009	Total
Maternal age (years)										
<34	213,123	197,153	188,784	176,771	173,653	170,229	161,498		155,824	1,437,035
>34	30,678	30,163	30,716	32,250	33,473	35,228	36,962		38,665	268,135
Parity										
First	237,248	221,342	213,512	203,221	201,403	199,462	192,864		188,785	1,657,837
Second	6,325	5,757	5,785	5,678	5,588	5,857	5,473		5,554	46,017
Other	228	217	203	122	140	147	131		150	1,338
Gender										
Male	127,663	119,196	115,322	109,066	108,276	107,502	103,789		101,171	891,985
Female	116,055	108,049	104,104	99,897	98,802	97,904	94,630		93,273	812,714
Gestational age (weeks)										
<37	21,938	20,466	21,335	19,639	19,273	19,576	19,558		18,768	160,553
>37	221,863	206,850	198,161	189,382	187,856	185,890	178,909		175,720	1,544,631
Birth weight (kg)										
<1.5	3,528	3,299	3,448	3,286	3,277	3,333	3,289		3,225	26,685
1.5–2.5	18,433	16,817	16,581	15,809	15,371	15,638	15,042		14,922	128,613
>2.5	216,853	202,641	195,232	186,298	184,701	183,030	176,905		176,341	1,522,001

**Table 2:** Birth prevalence of cleft lip with or without cleft palate (CL/P), cleft palate (CP) and other congenital anomalies in newborns in Taiwan from 2002–2009.

	Years								
	2002	2003	2004	2005	2006	2007	2008	2009	Total Average
Total newborns	243,801	227,316	219,500	209,021	207,131	205,466	198,468	194,489	213,149
Normal newborn	241,526	225,404	217,507	207,172	205,492	203,852	196,980	193,148	211,385
All congenital anomalies	2,275	1,912	1,993	1,849	1,639	1,614	1,488	1,341	1,764
CL/P	271	232	270	202	171	180	182	154	208
(%)	0.11	0.10	0.12	0.10	0.08	0.09	0.09	0.08	0.10
CP	77	109	93	93	77	95	76	70	86
(%)	0.03	0.05	0.04	0.04	0.04	0.05	0.04	0.04	0.04
Other	1927	1571	1630	1554	1391	1339	1230	1117	1,476
(%)	0.85	0.82	0.82	0.84	0.85	0.83	0.83	0.83	0.84

We then categorized the information into two periods, 2002–2005 and 2006–2009, to determine the demographic characteristics related to prevalence of CL/P and CP, including mothers’ age, parity, gender, gestational age, and birth weight. Results from analysis are shown in Table 3. Higher birth prevalence of CL/P or CP was associated with mothers aged ≥34 years, parity (being the second or other pregnancy), being male for CL, being female for CP, lower gestational age at birth (≤37 weeks) and lower birth weight (<1.5 kg). Greater gestational age was observed with lower birth prevalence of facial cleft deformity, particularly in the earlier cohort. For those born in 2002–2005, 0.4% and 0.06% of those with CL/P and CP, respectively, were ≤37 weeks at birth compared to 0.4% and 0.08% of those in the later cohort. For both cohorts, most of those with CL/P or CP weighed <1.5 kg at birth. (Table 3).

**Table 3:** The distribution of cleft lip with or without cleft palate (CL/P) and cleft palate (CP) in newborns in Taiwan during 2002–2005 and 2006–2009.

		Normal newborns				CL/P				CP			
		2002–2005		2006–2009		2002–2005		2006–2009		2002–2005		2006–2009	
		n	%	N	%	n	%	n	%	n	%	n	%
Maternal age (years)													
	≥34	769,328	86.3	656,659	82.1	844	0.1	538	0.1	321	0.04	257	0.04
	<34	122,281	13.7	142,792	17.9	131	0.1	148	0.1	51	0.04	61	0.04
Parity													
	first	867,730	97.3	776,685	97.2	926	0.1	662	0.1	357	0.04	310	0.04
	second	23,129	2.6	22,224	2.8	46	0.2	23	0.1	14	0.06	8	0.04
	other	750	0.1	563	0.07	3	0.4	2	0.4	1	0.1	0	0.00
Gender													
	male	466,687	52.4	417,309	52.2	564	0.1	411	0.1	169	0.04	117	0.03
	female	424,714	47.7	382,003	47.8	406	0.1	271	0.1	203	0.05	201	0.05
Gestational age (weeks)													
	<37	80,091	9.0	74,399	9.3	303	0.4	267	0.4	47	0.06	58	0.08
	≥37	811,514	91.0	725,069	90.7	672	0.1	420	0.1	325	0.04	260	0.04
Birth Weight (kg)													
	<1.5	11,365	1.3	11,089	1.4	213	1.9	201	1.8	20	0.2	21	0.2
	1.5–2.5	66,424	7.6	60,110	7.6	127	0.2	89	0.2	51	0.1	49	0.1
	>2.5	796,506	91.1	717,845	91.0	628	0.1	391	0.1	295	0.04	243	0.03

Logistic regression analysis of the correlation between CL/P, CP and cohort demographic characteristics (Table 4) showed that, for newborns with CL/P, the odds of prevalence was significantly lower in females than in male newborns (0.8, 95% CI: 0.7–0.8, P<0.0001), lower in newborns with gestational age >37 weeks than those born earlier (0.2, 95% CI: 0.18–0.22, P<0.0001), higher with the second parity than with the first born or other parity (1.6, 95% CI: 1.2–2.0, P = 0.0004 and 3.9, 95% CI: 1.6–9.4, P = 0.002, respectively), and lower in newborns with birth weight 1.5–2.5 kg and >2.5 kg than in those with birth weight <1.5 kg (0.09, 95% CI: 0.08–0.1, P<0.0001 and 0.04 95% CI: 0.03–0.04, P<0.0001, respectively). For newborns with CP, the odds of prevalence was significantly higher in females than in males (1.5, 95% CI: 1.3–1.8, P<0.0001), lower in newborns with gestational age >37 weeks than those with gestational age <37 weeks (0.6, 95% CI: 0.5–0.7, P = 0.04) and lower in newborns with birth weight 1.5–2.5 kg and >2.5 kg than those with birth weight <1.5 kg (0.4, 95% CI: 0.3–0.6, P<0.0001 and 0.2, 95% CI: 0.1–0.3, P<0.0001, respectively). (Table 4)

**Table 4:** Logistic regression analysis of the distribution of cleft lip with or without cleft palate (CL/P) and cleft palate (CP) in newborns in Taiwan during 2002–2009 after adjusting for time.

	CL/P			CP		
	OR	CI	P value	OR	CI	P value
Maternal age (years)						
<34	Ref	–	–	Ref	–	–
≥34	1.1	1.0–1.28	0.08	1.1	0.9–1.3	0.6
Parity						
First	Ref	–	–	Ref	–	–
Second	1.6	1.2–2.0	0.0004*	1.2	0.8–1.8	0.4
Other	3.9	1.6–9.4	0.002	1.9	0.3–13.3	0.5
Gender						
Male	Ref	–	–	Ref	–	–
Female	0.8	0.7–0.8	<0.0001*	1.5	1.3–1.8	<0.0001*
Gestational age (weeks)						
<37	Ref	–	–	Ref	–	–
≥37	0.2	0.2–0.2	<0.0001*	0.6	0.5–0.7	<0.0001*
Birth weight (kg)						
<1.5	Ref	–	–	Ref	–	–
1.5–2.5	0.09	0.08–0.1	<0.0001*	0.4	0.3–0.6	<0.0001*
>2.5	0.04	0.03–0.04	<0.0001*	0.2	0.1–0.3	<0.0001*

* indicates statistical significance.

## Discussion

Our study characterizes the current national birth prevalence of CL/P and CP in Taiwan. We found overall birth prevalence of 0.1% for CL/P and 0.04% for CP over this 8-year period. Previous studies showed a worldwide prevalence of 0.1% for CL/P [15]. While Asian populations generally have the highest rates, rates vary widely, reflecting genetic, environmental and health equality factors [1]. A study in Jordan of 25,440 infants reported incidence of facial cleft deformities to be 0.2% [15]. Other studies report varying rates in Asian populations, from a low of 0.06 % in Japanese infants born in California [16] to 0.2% in a one-year national study in Korea [2] and 0.2% in a study from mainland China [3]. Our overall prevalence rate of 0.1% was lower than those in these studies, although each involved different methodologies, which could explain differences in rates. While these studies used sampling methods to estimate birth rates of cleft deformities, our study employed a national database of information obtained at birth in the hospital, and therefore should be considered accurate as a birth rate. Interestingly, the rates of both cleft deformities and births fell during our study period, from the first cohort to the later cohort. We are unable to account for this trend, although we suspect it may be related to an exceptionally high rate of first births for all mothers, as well as the gradual acceptance of and increase in abortion after its legalization in the early 2000 s. Another study in Ireland found no change in rates over time when comparing 5-year cohorts from 1981 to 2000 [17]. However, in the Netherlands, a similar pattern of falling rates for facial cleft deformations during 1997–2006 was linked to prenatal identification of congenital anomalies [18]. A 2011 study in India reported an apparent falling rate of cleft deformities, but offered no explanation for this trend [7].

In our study, CL/P was more frequently found in and associated with male newborns. In addition to higher prevalence of cleft deformities, boys suffer greater psychosocial disruption from facial cleft deformities than girls [11]. In the US, the prevalence of CP is 0.04 per 1,000 and CP is more frequently found in female infants [19]. Our study also showed that more female newborns had CP than males. This is in accord with most studies reporting higher incidence of CL/P in males [2], [3], [17], [20], [21] and higher incidence of CP in girls [2], [7], [17], [20], [22]. One study of 45,676 discharges for cleft deformities in children in the US from 1997–2007 found no sex differences in rates [23]. This study, however, did not track birth or longitudinal diagnosis, but only children receiving surgical correction of facial cleft deformities. As the authors noted, children with only mild malformation or those without health insurance may not have presented for treatment [23].

In the present study, mothers with age ≥34 years were associated with an increased risk of having infants with a facial cleft deformity, but the association did not reach statistical significance. Other studies report similar results involving older mothers or parents [6], [21], [22]. A 5-year study in Texas of 1.8 million live births also found that mothers >40 years were more likely to give birth to those with congenital facial cleft deformities [22]. A study in Estonia showed that 28.4% of mothers and 37.7% of fathers who had children with congenital facial cleft deformities were <30 years. These mothers also had high rates of psychological stress, physical trauma (e.g., accidents, being hit by an animal, domestic violence), one or more medical abortions and exposure to teratogenic toxic substances [21]. Advanced maternal age, then, is likely associated with yet-unidentified factors. We also found that, for 94% or more of all live births, with or without deformity, the infants were recorded as the mothers’ first birth and they were associated with statistically lower prevalence of CL/P. While few studies of facial cleft deformity track parity, the Texas study found that children who were the second or higher birth accounted for 29.2% of births, but 31.6% of CP and 33.7% of CL/P [22]. Undoubtedly, these mothers were older, and other factors may also have accounted for this higher prevalence.

We found that gestational age <37 weeks was associated with greater birth prevalence of facial cleft deformity, especially in the earlier cohort. Association of facial cleft disorders with low birth weight, premature birth or other developmental anomalies has led to calls for future research in prevention intervention and identifying genetic risk and predisposition [1]. In terms of the decreasing numbers of infants with of facial cleft deformities associated with greater gestational age in the later cohort, we speculate that prenatal identification of congenital anomalies (which could include facial clefts) may have led to elective pregnancy termination. As the secondary database tracked only live births, we cannot ascertain the effect of early detection on birth rates. However, decreased prevalence of CL/P but not CP was observed in the later cohort, and this may represent the increasingly acceptable effect of legalized elective termination in the early 2000 s based on visible CL/P via ultrasound evaluation.

In Taiwan, low birth weight of <1.5 kg was also significantly associated with facial cleft deformities. While birth weight is often not recorded in these studies, many find associated pregnancy complications (twins, gestational diabetes) or other genetic anomalies in these infants, perhaps diagnosed prenatally [3], [6], [15]. Newborns with very low birth weight may have had some fatal genetic anomaly, died at birth or were stillborn (and thus were not counted) or were electively aborted. As an earlier study at a regional hospital in Taiwan noted, prenatal genetic screening is routinely available and has been accompanied by a sharp drop in the prevalence of Down’s syndrome [6]. The importance of gestational age and birth weight in infants with facial cleft deformities warrants further study. Characterizing the rates of facial cleft deformities may allow resource allocation to ensure physician counseling and timely treatment, which have been shown to reduce facial cleft deformity rates and accompanying morbidity [1], [7].

Our study has certain limitations. The database did not provide information on maternal health or peri-conceptual conditions, which may play a role in development of facial cleft deformities [1], [3], [21]. Additional demographic factors such as socioeconomic status and area of residence (urban or rural), which have been associated with different prevalence rates [1], [22], were also not recorded. We also did not capture data of those children diagnosed later than at birth. Most diagnoses of CL/P or CP are made by age three [17], [23]. Our numbers therefore represent a conservative under-reporting of the actual prevalence; some congenital anomalies recorded in the secondary database were far less than thos expected for occurrence of major congenital anomalies (e.g., neural tube defect, 50%).

In conclusion, overall prevalence for congenital cleft deformities in Taiwan was 0.1%, in keeping with high rates in Asia. Results of this study suggest the need for increased physician awareness, early identification of and possibleearly intervention for high risk mothers aged ≥34 years, those with multiple parities and those whose newborn weighed <1.5 kg at birth, which may be a potential strategy to counter long-term adverse effects on speech and language in this population.
